# Efficacy and Safety Analyses of Recombinant Factor VIIa in Severe Post-Partum Hemorrhage

**DOI:** 10.3390/jcm13092656

**Published:** 2024-05-01

**Authors:** Camila Caram-Deelder, Hellen McKinnon Edwards, Jarmila A. Zdanowicz, Thomas van den Akker, Camilla Birkegård, Jan Blatný, Johanna G. van der Bom, Giuseppe Colucci, Derek van Duuren, Nan van Geloven, Dacia D. C. A. Henriquez, Marian Knight, Lars Korsholm, Andrea Landorph, Géraldine Lavigne Lissalde, Zoe K. McQuilten, Daniel Surbek, Cameron Wellard, Erica M. Wood, Frederic J. Mercier

**Affiliations:** 1 Leiden University Medical Center, 2333 Leiden, The Netherlands; 2 Department of Obstetrics and Gynaecology, Copenhagen University Hospital Herlev, 2730 Herlev, Denmark; 3 Department of Obstetrics and Gynecology, Inselspital, Bern University Hospital, University of Bern, 3010 Bern, Switzerland; 4 Athena Institute, Vrije Universiteit, 1081 Amsterdam, The Netherlands; 5 Novo Nordisk A/S, 2860 Søborg, Denmark; 6 Department of Paediatric Oncology, University Hospital Brno, and Masaryk University, 625 00 Brno, Czech Republic; 7 Clinica Sant’Anna, 6924 Sorengo, Switzerland; 8 University of Basel, 4001 Basel, Switzerland; 9 Amsterdam University Medical Center, University of Amsterdam, 1105 Amsterdam, The Netherlands; 10 National Perinatal Epidemiology Unit, Nuffield Department of Population Health, University of Oxford, Oxford OX3 7LF, UK; 11 Department of Hematology, University Hospital, 30900 Nîmes, France; 12 Transfusion Research Unit, Department of Epidemiology and Preventive Medicine, School of Public Health and Preventive Medicine, Monash University, Melbourne 3004, Australia; 13 Department of Haematology, Monash Health, Melbourne 3004, Australia; 14 Hôpital Antoine Béclère, Assistance Publique–Hôpitaux de Paris, Université Paris Saclay, 92140 Clamart, France

**Keywords:** recombinant activated factor VII, post-partum hemorrhage, pregnancy complications, hematologic, coagulants, therapeutic use, thromboembolic events, delivery, obstetric, maternal mortality

## Abstract

**Background**: Despite a range of available treatments, it is still sometimes challenging to treat patients with severe post-partum hemorrhage (sPPH). Objective: This study evaluated the efficacy and safety of recombinant activated factor VIIa (rFVIIa) in sPPH management. **Methods**: An open-label, multi-center, randomized controlled trial (RCT; NCT00370877) and four observational studies (OS; OS-1 (NCT04723979), OS-2, OS-3, and OS-4) were analyzed regarding efficacy (need for subsequent invasive procedures, including uterine compression sutures, uterine or iliac artery ligations, arterial embolization, or hysterectomy) and safety (incidence of thromboembolic events (TE) and maternal mortality) of rFVIIa for sPPH. The RCT, and OS-1 and OS-2, included a control group of women who did not receive rFVIIa (with propensity score-matching used in OS-1 and OS-2), whereas OS-3 and OS-4 provided descriptive data for rFVIIa-exposed women only. **Results**: A total of 446 women exposed to rFVIIa and 1717 non-exposed controls were included. In the RCT, fewer rFVIIa-exposed women (50% [21/42]) had an invasive procedure versus non-exposed women (91% [38/42]; odds ratio: 0.11; 95% confidence interval: 0.03–0.35). In OS-1, more rFVIIa-exposed women (58% [22/38]) had an invasive procedure versus non-exposed women (35% [13.3/38]; odds ratio: 2.46; 95% confidence interval: 1.06–5.99). In OS-2, 17% (3/18) of rFVIIa-exposed women and 32% (5.6/17.8) of non-exposed women had an invasive procedure (odds ratio: 0.33; 95% confidence interval: 0.03–1.75). Across all included women, TEs occurred in 1.5% (0.2% arterial and 1.2% venous) of rFVIIa-exposed women and 1.6% (0.2% arterial and 1.4% venous) of non-exposed women with available data. **Conclusions**: The positive treatment effect of rFVIIa on the RCT was not confirmed in the OS. However, the safety analysis did not show any increased incidence of TEs with rFVIIa treatment.

## 1. Introduction

Post-partum hemorrhage (PPH) is a significant cause of maternal morbidity worldwide, with potential complications including loss of fertility and death [1]. PPH is estimated to occur in 6–11% of all deliveries, with severe PPH (sPPH) affecting 1–3% [2,3]. Although treatment guidelines vary, uterotonics are recommended prophylactically as well as for PPH treatment [1,4,5]. If bleeding persists, depending on the underlying cause, options may include minimally invasive obstetrical interventions (e.g., manual removal of the placenta/manual exploration of the uterus and/or episiotomy/genital tract sutures), fluid replacement, hemostatic agents, and blood product transfusions [1,2,4]; and non-invasive/minimally invasive mechanical methods (e.g., manual uterine compression/intra-uterine balloon tamponade) [1,2]. If bleeding remains uncontrolled, conservative invasive procedures may be considered, including selective arterial embolization, laparotomy, uterine/iliac artery ligation, or uterine compression sutures [1]. Life-threatening situations may require emergency hysterectomy [4]. Risks associated with these invasive procedures include wound infection, vessel damage, sepsis, thromboembolic events (TEs), organ failure, loss of reproductive potential, and adverse psychological/social impacts [6,7,8,9,10]. 

Despite the multitude of options described above, there remains an unmet clinical need in sPPH management. Effective and preferably non-invasive treatments are desirable to minimize the need for surgical interventions, avoid future infertility, and reduce maternal mortality. The off-label use of recombinant activated human factor VII (rFVIIa) is recommended in some sPPH management guidelines, typically at a dosage of 60–90 µg/kg, although recommended timing varies (usually, as a last resort, before hysterectomy or in life-threatening situations) [11,12,13,14]. Data regarding rFVIIa treatment of sPPH have been reported; however, it is unclear whether the benefits outweigh the risks [15,16,17,18,19]. Concerns regarding TEs remain, as the majority of studies involving rFVIIa were carried out in non-obstetric populations [20]. Therefore, it has not yet been possible to establish a benefit–risk profile for this treatment in sPPH.

Here, we describe a collaborative project that aimed to evaluate the efficacy and safety of rFVIIa for sPPH management. Data from a previously published open-label randomized controlled trial (RCT) [17] were re-analyzed alongside four observational studies (OS). Although some results from these studies have previously been published [17,18,21,22,23,24], in order to provide information on a more clinically relevant endpoint and an updated safety analysis, datasets were re-analyzed at the individual patient level using a common primary endpoint and propensity score-matching strategies where appropriate (OS-1 and OS-2).

## 2. Materials and Methods

### 2.1. Datasets

In the multi-center, open-label, parallel-group RCT (NCT00370877), women with sPPH were randomized 1:1 to rFVIIa treatment or standard-of-care following the failure of sulprostone to control bleeding. The RCT was conducted at seven sites in France and one site in Switzerland, and originally assessed the reduction in the requirement for invasive second-line therapies in patients with sPPH (Table 1; Supplementary Materials Section S1.1; Table S1) [17].

Of the four observational studies (PPH consortium (Denmark, The Netherlands, and the UK; NCT04723979; OS-1), Bern University Hospital (OS-2), the UniSeven registry (Czech Republic; OS-3), and the Australian and New Zealand Haemostasis Registry (ANZHR; OS-4)), OS-1 and OS-2 provided data for rFVIIa-exposed and non-exposed women, and OS-3 and OS-4 provided data for rFVIIa-exposed women only. Inclusion criteria, sPPH definition, and rFVIIa use varied amongst the studies (Table 1; Supplementary Materials Section S1.1; Table S1). Relevant ethical approvals and consent were obtained as required and stated at the end of the manuscript.

### 2.2. Patient Populations

Three populations were defined for this combined analysis. The full analysis set included all randomized women for the RCT and all women who met the inclusion criteria of the observational studies (Table 1). The full analysis set was used for efficacy analyses for the RCT and for assessing safety in all studies. Secondly, a sub-population of women “at risk of further invasive procedures” was defined in the observational studies.

The third patient population was the propensity score analysis set (PSAS). Due to differences between women who were/were not exposed to rFVIIa in OS-1 [23] and OS-2, propensity score-matching was used to construct two comparable subgroups. As the number of comparable controls (non-exposed women) available per rFVIIa-exposed woman varied, a maximum of 4 controls were selected. To counteract numerical imbalance, the controls were weighted according to the number of controls for each exposed woman. 

### 2.3. Endpoints

The primary endpoint was occurrence of invasive procedures, including uterine compression sutures, uterine or iliac artery ligations, radiological arterial embolization, or hysterectomy (timeframes provided in Supplementary Materials Section S1.2, Figure S1). Secondary endpoints included amount of blood products transfused (including red blood cells (RBCs) and fresh frozen plasma (FFP)) and occurrence of hysterectomy. Safety endpoints included occurrence of venous TEs (VTEs), arterial TEs (ATEs), and maternal deaths (timeframes provided in Supplementary Materials Section S1.3).

### 2.4. Statistical Analysis

For the RCT, the primary endpoint was compared between rFVIIa and reference (non-exposed) groups in the full analysis set according to the odds ratio (OR) and relative risk reduction (with 95% confidence intervals (CI) and *p*-value calculated from a two-sided chi-square test (5% significance level)). 

For the comparative analysis of OS-1 and OS-2, the balance of confounding variables was checked to ensure the propensity score model had been specified correctly (Supplementary Materials Section S1.4). An exact conditional logistic regression was used for the comparison between rFVIIa-exposed and non-exposed women for the primary and secondary endpoints. The test assessing the OR was two-sided (5% significance level) and no multiplicity adjustment was required as the analysis was based on only one primary endpoint. In OS-3 and OS-4, a descriptive analysis of the primary endpoint was conducted on the population at risk.

Safety endpoints were analyzed descriptively. Meta-analyses were conducted on the occurrence of TEs in rFVIIa-exposed (all studies) and non-exposed women (RCT, OS-1, and OS-2) using a generalized linear mixed model to estimate proportions, based on the binomial distribution using a logit link function with study included as a random effect.

## 3. Results

### 3.1. Patient Populations and Baseline Characteristics

Across all studies, 446 women were exposed to rFVIIa, with 1717 women notexposed. The RCT full analysis set included 42 women randomized to early treatment with rFVIIa (≤60 min of sulprostone administration) and 42 to the reference group. Nine women in the reference group also received rFVIIa (eight on a compassionate use basis, one in error). The full analysis set of the four observational studies included 395 rFVIIa-exposed and 1684 non-exposed women; and the overall full analysis set for all five studies comprised 437 rFVIIa-exposed and 1726 non-exposed women (Table 2). Two-hundred and eighteen rFVIIa-exposed women were included in the population at risk of invasive procedures across all observational studies. The PSAS of OS-1 included 40 rFVIIa-exposed women and 115 matched controls. Two of the 40 rFVIIa-exposed women and 4/115 matched controls were excluded from the primary analyses due to hysterectomy during the 20 min lag period, and 3/115 matched controls were excluded due to having been matched to the rFVIIa-exposed women who had a hysterectomy in this time period. The PSAS of OS-2 included 18 rFVIIa-exposed women and 43 matched controls (one of whom was excluded from the primary analysis due to a hysterectomy during the lag period). Full details of the outcomes of the propensity score-matching process are presented in the Supplementary Materials Section S2.1; Table S2 and Figure S2.

There were some variations in the baseline characteristics in the full analysis set between studies, including mode of delivery and primary cause of PPH (Table 2), and clinical characteristics of rFVIIa-exposed and matched controls in the PSAS were well-balanced (Table S2). In the RCT, one woman had an invasive procedure prior to rFVIIa exposure, whereas in the full analysis set of the observational studies, the proportion of women with a prior invasive procedure ranged from 23% (UK cohort of OS-1) to 62% (OS-2). In the OS-1 and OS-2 PSASs, none of the women previously had a hysterectomy, as per the study design.

### 3.2. Efficacy of rFVIIa for the Treatment of sPPH (Comparative Studies)

#### 3.2.1. Randomized Controlled Trial

**Figure 1:**
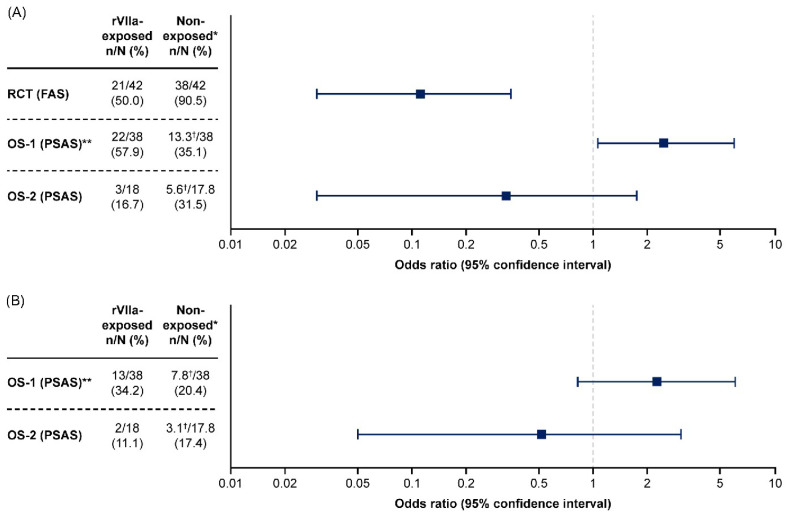
Odds of (**A**) any invasive procedure or (**B**) hysterectomy among rFVIIa-exposed women compared with non-exposed women in the randomized controlled trial, OS-1 and OS-2. The occurrence of (**A**) any invasive procedures or (**B**) hysterectomy after randomization in the RCT, after rFVIIa administration in rFVIIa-exposed patients in the OS, and after time of propensity score-matching in matched control patients from the OS. Odds ratio was not pre-specified for the hysterectomy endpoint for the RCT, and so is not available for inclusion in Panel B. The x-axes in the figures use a log scale. * Includes patients from the reference group of the RCT and matched controls from OS-1 and OS-2. ** The propensity score analysis set of OS-1 included patients from Denmark and The Netherlands. ^†^ The number of matched control patients in these groups was not a whole number due to the weighting that was used for the matching process. Data for the non-exposed groups of OS-1 and OS-2 were based on 108 and 42 women, respectively (women from the PSAS with hysterectomy prior to matching time were excluded from this analysis). OS-1, PPH Consortium; OS-2, Bern University Hospital Study. FAS, full analysis set; OS, observational study; PPH, post-partum hemorrhage; PSAS, propensity score-matched analysis set; RCT, randomized controlled trial; rFVIIa, recombinant activated factor VII.

For the primary endpoint of occurrence of invasive procedures, 21/42 (50%) rFVIIa-exposed women had a subsequent invasive procedure compared with 38/42 (91%) in the reference group, corresponding to a 45% relative reduction in risk (95% CI: 0.24–0.60; *p* < 0.0001) of an invasive procedure in the rFVIIa group. The OR between the two groups was 0.11 (95% CI: 0.03–0.35; Table 3 and Figure 1).

A post hoc subgroup analysis of this endpoint showed that, amongst women with a baseline fibrinogen plasma level ≥ 2 g/L, invasive procedures occurred in 33% (9/27) of rFVIIa-exposed women versus 94% (31/33) of those in the reference group. For women with a baseline fibrinogen plasma level < 2 g/L, invasive procedures occurred in 88% (7/8) of rFVIIa-exposed women versus 100% (5/5) in the reference group (Supplementary Materials Section S2.2; Figure S3).

In the rFVIIa-exposed group, 3/42 (7%) women underwent a hysterectomy versus 8/42 (19%) in the reference group, corresponding to a 62.5% relative reduction in risk (95% CI: −0.32–0.89; *p* = 0.19) of a hysterectomy in the rFVIIa group (Table 3; odds ratio not pre-specified for this endpoint). Median duration of bleeding in the rFVIIa group was 115.0 min (interquartile range (IQR) 60.0–195.0) versus 177.5 min (IQR 130.0–250.0) in the reference group (no statistical testing applied). Units of RBCs and FFP transfused were similar between groups (Table S3).

#### 3.2.2. Observational Studies (OS-1 and OS-2)

For the primary endpoint of occurrence of invasive procedures in OS-1, 22/38 (58%) rFVIIa-exposed women had a subsequent invasive procedure compared with 13.3/38.0 (35%) in the weighted matched control group (conditional OR: 2.46; 95% CI: 1.06–5.99, *p* = 0.04; Table 3 and Figure 1). In OS-2, invasive procedures occurred in 3/18 (17%) of rFVIIa-exposed women and 5.6/17.8 (32%) of the weighted matched control group (conditional OR: 0.33; 95% CI: 0.03–1.75, *p* = 0.27; Table 3 and Figure 1). Of note, the denominator of the control percentage is based on data from 108 women for OS-1 and 42 women for OS-2, and weighted according to the number of controls within pairs (Supplementary Materials Sections S1.4 and S2.1). A sensitivity analysis performed for OS-1 and OS-2 to account for the fact that some matched control patients received rFVIIa at a later time point yielded similar results (Supplementary Materials Section S2.2; Table S4).

In the rFVIIa-exposed group of OS-1, 13/38 (34%) women had a hysterectomy versus 7.8/38 (20%) in the reference group (OR: 2.23; 95% CI: 0.83–6.06; *p* = 0.12) (Table 3 and Figure 1). In OS-2, 2/18 (11%) rFVIIa-exposed women had a hysterectomy compared with 3.1/17.8 (17%) in the weighted reference group (OR: 0.52; 95% CI: 0.05–3.03; *p* = 0.68).

In OS-2, median duration of bleeding (from onset to stop of sPPH) for women in the PSAS was 167.5 min (IQR: 101.0–235.0 min) for matched-exposed women and 250.0 min (IQR: 138.0–673.5 min) for matched controls. Details of duration of bleeding were unavailable for OS-1. In the PSASs of both OS-1 and OS-2, mean volumes of RBCs and FFP before and after matching time were comparable between rFVIIa-exposed women and matched controls (Table S3).

### 3.3. Clinical Outcomes following rFVIIa Treatment of sPPH (Non-Comparative Studies)

In the observational studies without a comparator arm (OS-3 and OS-4), 23% (10/43) and 30% (22/74) of women in the population at risk had an invasive procedure following rFVIIa exposure, respectively. For the secondary endpoint of hysterectomy following rFVIIa administration, 21% (9/43) of rFVIIa-exposed women in OS-3 and 20% (15/76) of exposed women in OS-4 went on to have a hysterectomy.

### 3.4. Safety of rFVIIa in the Management of sPPH

A total of 446 women across all studies were exposed to rFVIIa, including nine women in the RCT reference group. VTEs were reported in 2/51 rFVIIa-exposed and in none of the 33 non-exposed women in the RCT, with no ATEs reported in either group (Table 4).

In the observational studies, VTEs were reported in 3/358 rFVIIa-exposed women (none were fatal). Additionally, one VTE occurred in the Dutch cohort of OS-1; however, TEs in this cohort were only reported if the patient underwent an embolization procedure (which was the case for 23 exposed women and 144 non-exposed women), and these results are therefore presented separately. Data were missing for nine non-exposed women in the Danish cohort of OS-1. A VTE was reported in 7/452 non-exposed women (from OS-2 and the Danish and UK cohorts of OS-1), and two VTEs were reported in the Dutch cohort of OS-1. An ATE was reported in 1/358 rFVIIa-exposed women across the observational studies (myocardial infarction, OS-4 (fatal)) and in 1/452 non-exposed women. One woman experienced an ATE in the non-exposed group of the Dutch cohort of OS-1 (Table 4). Further details on all TEs are provided in the Supplementary Materials Section S2.3.

**Figure 2:**
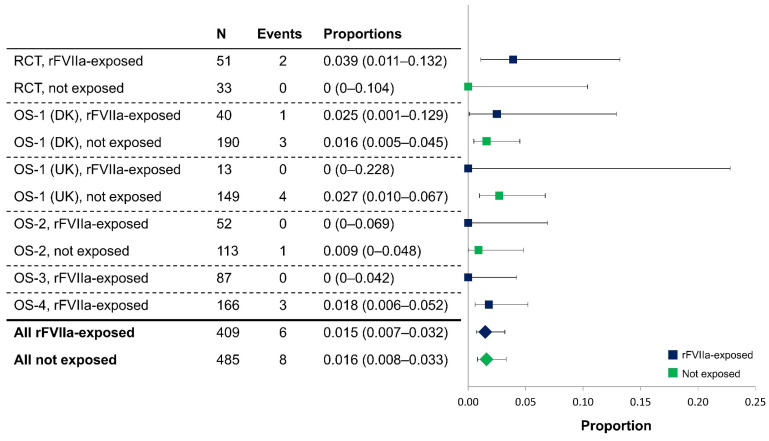
Meta-analysis of thromboembolic events in rFVIIa-exposed and non-exposed women in the randomized controlled trial and observational studies (full analysis set, excluding patients with unavailable data). In the Dutch cohort of OS-1, a TE was only recorded if it was a complication of an embolization; therefore, TE data from this cohort were excluded from the meta-analysis. OS-1, PPH Consortium; OS-2, Bern University Hospital Study; OS-3, UniSeven; OS-4, Australian and New Zealand Hemostasis Registry. DK, Denmark; OS, observational studies; PPH, post-partum hemorrhage; RCT, randomized controlled trial; rFVIIa, recombinant activated factor VII; TE, thromboembolic event; UK, United Kingdom.

The results of a meta-analysis showed the proportion of women with a TE for all studies was 1.5% in rFVIIa-exposed women versus 1.6% in non-exposed (Figure 2). The overall proportions of women with an ATE or VTE were comparable between groups (0.2% for ATEs in both groups, and 1.2% versus 1.4% for VTEs in exposed and non-exposed women, respectively).

Fifteen deaths were reported in 446 women exposed to rFVIIa across the studies, with nine deaths reported among the 1717 women in the reference groups (Table S5). The cause of death for 9/15 exposed women was not related to a TE and unknown or not available for four women; the remaining two exposed women had experienced a TE, but the death was assessed as unlikely to be related to rFVIIa by a study clinician (see the Supplementary Materials Section S2.3 for further details).

### 3.5. rFVIIa Dosing and Timing of Administration

**Figure 3:**
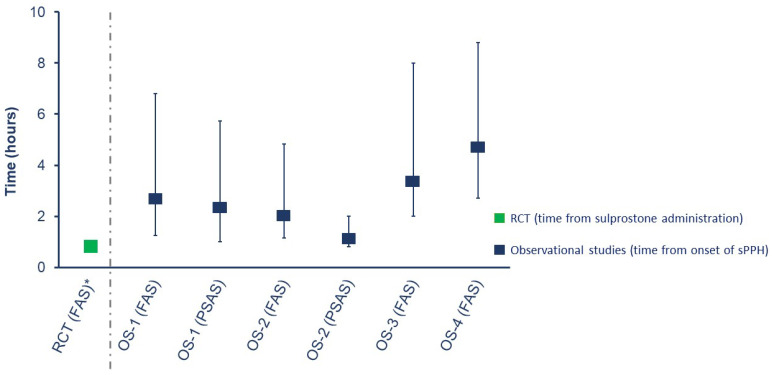
Median time from onset of sPPH to administration of first dose of rFVIIa in the randomized controlled trial and observational studies (full analysis or propensity score analysis sets). Bars show interquartile range. Data shown for the OS-1 FAS are from Denmark and The Netherlands only. * In the RCT, women were randomized if sulprostone had failed to control bleeding within 1 h of administration; exact timing between sPPH onset and sulprostone administration was not available. OS-1, PPH Consortium; OS-2, Bern University Hospital Study; OS-3, UniSeven registry; OS-4, ANZHR; ANZHR, Australian and New Zealand Hemostasis Registry; FAS, full analysis set; OS, observational study; PPH, post-partum hemorrhage; PSAS, propensity score analysis set; RCT, randomized clinical trial; rFVIIa, recombinant activated factor VII; sPPH, severe post-partum hemorrhage.

In the RCT, women received rFVIIa within 60 min of sulprostone administration. In the full analysis set of the observational studies, the median time (IQR) from onset of sPPH to first dose of rFVIIa varied, ranging from 127.5 min (71.0−290.5) in OS-2 to 291.0 min (160.0–525.0) in OS-4 (Figure 3). Details of doses/dosage received in the studies are provided in the Supplementary Materials Section S2.4; Figures S4 and S5.

## 4. Discussion

In this collaborative project, data regarding the use of rFVIIa in over 400 women with sPPH across one RCT and four observational studies were analyzed in parallel, allowing for the collation of efficacy and safety data from multiple international sources. Data analysis revealed contrasting results for the primary endpoint of occurrence of invasive procedures across the studies with a comparator arm. In the RCT, there was a reduction in invasive procedures in rFVIIa-exposed women compared with the reference group. In contrast, the results from OS-1 show that more rFVIIa-exposed women underwent an invasive procedure compared with non-exposed PS-matched controls; and in OS-2, there was no statistically significant difference between groups. Nevertheless, the safety analysis did not show any increased incidence of TEs with rFVIIa treatment.

There is a relevant safety concern of development of TEs following the use of rFVIIa in sPPH, due to a potential overstimulation of the coagulation system [25]. Previously, a Cochrane review found a significant increase in ATEs with rFVIIa treatment of bleeding in patients without hemophilia [25]; and women with sPPH may have an increased risk of developing TEs [26]. The current analysis showed that proportions of TEs were similar in women with sPPH exposed to rFVIIa and non-exposed women, with the majority of events being VTEs. Of note, 13/15 deaths in rFVIIa-exposed women were recorded during OS-4, in which the median time from onset of sPPH until the first administration of rFVIIa was just under 5 h, with some of these women having been transferred from a local center (often in a remote location) before treatment. Real-world data present challenges for analysis, and the contrasting efficacy results found in the RCT, OS-1, and OS-2 may have been due to residual confounding effects, such as the severity of bleeding when rFVIIa was administered.

Although it is not possible to definitively conclude why the efficacy outcomes from the comparative studies varied, there are some potential hypotheses that can be considered. The studies were diverse in terms of design, patient populations, and setting, with data collected through a variety of sources. Clinical experience from previously approved indications of rFVIIa suggested timing of administration may be critical, with earlier use potentially being more beneficial [27]. The European Medicines Agency approval of rFVIIa authorizes its use as a treatment for sPPH after failure of uterotonics [28], and it is likely that optimal timing of administration may depend on clinical circumstances, such as necessity for surgical repair of trauma or transfer to a larger treatment center.

As coagulopathy was not evaluated in this project, no conclusions could be drawn regarding its impact on the clinical efficacy of rFVIIa treatment in this setting. Further research is necessary to investigate in which type of patient and within which timeframe rFVIIa would be relevant to treat sPPH.

There are some limitations of this project that should be considered when interpreting the results. Since a limited number of variables could be included in the PS-matching models for OS-1 and OS-2, it is possible that PS-matching did not remove all confounding, leading to under- or over-estimation of a possible effect of rFVIIa. As previously discussed [17], the RCT was a multi-center, open-label trial with a relatively small sample size, therefore confounding, observer bias, or random (false-positive) error cannot be excluded. Women were treated at referral centers with facilities available for the active management of PPH, which may have resulted in a higher likelihood of performing an invasive procedure within the comparator arm (no concurrent treatment), as some intervention was required to stop the hemorrhage. However, median times from randomization to invasive procedure initiation were similar between the two groups, and the level of reduction of invasive procedures with rFVIIa was fairly substantial [17]. Another consideration is that the treatment landscape has changed since these sPPH events occurred, with some treatments (intrauterine balloon, fibrinogen replacement, and tranexamic acid), and rapid bedside coagulation assessment more widely used today [29,30]. Therefore, these results may not fully correspond with the current treatment landscape.

## 5. Conclusions

In order to establish the efficacy and safety of a drug, ideally the highest possible evidence is required from an adequately powered double-blinded, randomized, placebo-controlled trial. Although such data are currently unavailable regarding the use of rFVIIa in sPPH, in our collaborative project, we collected globally available information and analyzed and presented it systematically. Our main finding from the safety meta-analysis indicated there was unlikely to be an increased incidence of either arterial or venous TEs associated with rFVIIa in women with sPPH. The multi-center, open-label RCT found a marked reduction in invasive procedures after rFVIIa treatment, whereas the results from the comparative observational studies did not confirm this. Going forward, more data regarding the clinical efficacy and safety of rFVIIa in different circumstances and causes of sPPH are desirable to optimize treatment.

## Figures and Tables

**Table 1:** Study design overview for the randomized controlled trial and observational studies.

Study Name	Randomized Controlled Trial	Observational Studies					
		OS-1			OS-2	OS-3	OS-4
		Denmark	The Netherlands	UK			
**Key inclusion criteria**	≥18 years; >27 weeks gestation; >1500 mL blood loss; sulprostone failure	≥10 U RBCs within 24 h	Obstetric hemorrhage: ≥4 U RBCs, or multicomponent blood transfusion *, or plasma in addition to RBCs	≥8 U RBCs within 24 h; ≥20 weeks of gestation	≥1500 mL blood loss within 24 h	≥1500 mL blood loss within 24 h	Obstetric hemorrhage with registered birth
**Definition of PPH**	Severe PPH: Blood loss >1500 mL measured in graduated bag and/or hemodynamically unstable and/or need for packed cells transfusion	Massive PPH: ≥10 U RBCs within 24 h	Persistent PPH: >1000 mL blood loss refractory to first-line interventions to control bleeding AND ≥1 of: ≥4 units of RBCs, multicomponent blood transfusion (RBCs and FFP and/or platelet concentrates), or plasma in addition to RBCs	Major PPH: ≥8 U RBCs within 24 h	Severe PPH: Continuous bleeding ≥1500 mL within 24 h	Severe PPH: Blood loss ≥1500 mL within 24 h	Obstetric case of hemorrhage with a registered delivery
**Protocol for rFVIIa** **administration**	60 ug/kg rFVIIa after sulprostone failure	–	–	–	rFVIIa at a dose of 60–90 μg/kg was administered **	–	–

* RBCs and fresh frozen plasma and or/platelet concentrates. ** Or at treating team’s discretion. OS-1, PPH Consortium; OS-2, Bern University Hospital Study; OS-3, UniSeven registry; OS-4, ANZHR, ANZHR, Australian and New Zealand Hemostasis Registry; FFP, fresh frozen plasma; h, hour; PPH, post-partum hemorrhage; RBCs, red blood cells; rFVIIa, recombinant activated factor VII; U, unit; UK, United Kingdom; –, not applicable.

**Table 2:** Patient characteristics in the randomized controlled trial and observational studies.

Study Name	Randomized Controlled Trial		Observational Studies									
			OS-1						OS-2		OS-3	OS-4
			Denmark		The Netherlands		UK					
Baseline Characteristics	rFVIIa N = 42	Ref N = 42	rFVIIa N = 40	No rFVIIa N = 199	rFVIIa N = 37	No rFVIIa N = 1223	rFVIIa N = 13	No rFVIIa N = 149	rFVIIa N = 52	No rFVIIa N = 113	rFVIIa N = 87	rFVIIa N = 166
**Age at delivery, years**												
N	–	–	40	199	37	1223	13	149	–	–	84	166
Median	–	–	33.0	33.0	31.0	32.0	34.0	33.0	–	–	31.5	33.0
IQR	–	–	29.0–38.0	30.0–36.0	28.0–34.0	28.0–35.0	28.0–36.0	29.0–36.0	–	–	28.0–36.0	29.0–37.0
Maternal body weight, kg *												
N	42	40	–	–	–	–	–	–	52	108	87	135
Median	68.0	70.0	–	–	–	–	–	–	70.0	72.5	72.0	65.0
IQR	62.0–76.0	60.0–79.0	––	––	––	–	–	–	61.0–83.0	67.0–82.0	66.0–80.0	55.0–75.0
Cause of PPH, n (%) **												
AIP ^†^	6 (14.3)	8 (19.0)	11 (27.5)	51 (25.6)	6 (16.2)	119 (9.7)	1 (7.7)	28 (25.5)	9 (17.3)	17 (15.0)	16 (18.4)	28 (16.9)
Placental abruption	–	–	4 (10.0)	13 (6.5)	0	12 (1.0)	1 (7.7)	14 (9.4)	5 (9.6)	8 (7.1)	9 (10.3)	15 (9.0)
Placental retention	4 (9.5)	1 (2.4)	4 (10.0)	21 (10.6)	2 (5.4)	217 (17.7)	–	–	1 (1.9)	31 (27.4)	1 (1.1)	–
Trauma ^‡^	7 (16.6)	2 (4.8)	7 (17.5)	50 (25.1)	3 (8.1)	89 (7.3)	1 (7.7)	26 (17.5)	1 (1.9)	9 (8.0)	6 (6.9)	5 (3.0)
Uterine atony	39 (92.9)	36 (85.7)	11 (27.5)	37 (18.6)	25 (67.6)	780 (63.8)	8 (61.5)	56 (37.6)	34 (65.4)	48 (42.5)	24 (27.6)	39 (23.5)
Other	–	–	3 (7.5)	27 (13.6)	1 (2.7)	6 (0.5)	2 (15.4)	14 (9.4)	2 (3.8)	0	32 (36.8)	87 (52.4)
Missing	0	0	0	0	0	0	0	1 (0.7)	0	0	14 (16.1)	24 (14.5)
**Delivery type, n (%)**												
Caesarean section	23 (54.8)	20 (47.6)	25 (62.5)	133 (66.8)	14 (37.8)	279 (22.8)	7 (53.9)	101 (67.8)	40 (76.9)	57 (50.4)	40 (46.0)	117 (70.5)
**Multiple birth (≥2), n (%)**												
Yes	7 (16.7)	7 (16.7)	1 (2.5)	12 (6.0)	3 (8.1)	71 (5.8)	3 (23.1)	4 (2.7)	8 (15.4)	15 (13.3)	6 (6.9)	8 (4.8)
**Invasive procedure(s) prior to rFVIIa, n (%)**												
Any	1 (2.4)	NA	23 (57.5)	NA	15 (40.5)	NA	3 (23.1)	NA	32 (61.5)	NA	21 (24.1)	63 (38.0)
Hysterectomy	0	NA	15 (37.5)	NA	6 (16.2)	NA	1 (7.7)	NA	3 (5.8)	NA	16 (18.4)	45 (27.1)

Full analysis set: rFVIIa-exposed women; N = 437; non-exposed women, N = 1726. * End of pregnancy weight, adjusted for weight of the baby. ** Primary cause of PPH: a woman may have had more than one cause of PPH. ^†^ In the RCT, AIP represents “placental insertion anomaly” captured as the cause of PPH in this trial. ^‡^ Trauma included all cases of trauma, uterine rupture, and birth canal injury. OS-1, PPH Consortium; OS-2, Bern University Hospital Study; OS-3, UniSeven registry; OS-4, ANZHR. AIP, abnormally invasive placenta; ANZHR, Australian and New Zealand Hemostasis Registry; IQR, interquartile range; NA, not applicable; PPH, post-partum hemorrhage; Ref, reference group; rFVIIa, recombinant activated factor VII; UK, United Kingdom; –, unavailable.

**Table 3:** Women with a subsequent invasive procedure or hysterectomy in the randomized controlled trial and comparative observational studies.

Study Name	Randomized Controlled Trial (FAS)		Observational Studies			
			OS-1 (PSAS) *		OS-2 (PSAS)	
Number of Women	rFVIIa N = 42	Ref N = 42	rFVIIa N = 38	Weighted Matched Controls N = 38 **	rFVIIa N = 18	Weighted Matched Controls N = 17.8 **
**At least one invasive procedure after rFVIIa administration ^†^** (primary endpoint), n (%)	21 (50.0)	38 (90.5)	22 (57.9)	13.3 ^‡^ (35.1)	3 (16.7)	5.6 ^‡^ (31.5)
**Odds ratio (95% CI)**	0.11 (0.03–0.35)		2.46 (1.06–5.99)		0.33 (0.03–1.75)	
*p*-value	–		0.04		0.27	
**Relative risk reduction,** **% (95% CI)**	44.7 (24–60)		NP		NP	
*p*-value	<0.0001		NP		NP	
**Women with** **hysterectomy,** **n (%)**	3 (7.1)	8 (19.1)	13 (34.2)	7.8 ^‡^ (20.4)	2 (11.1)	3.1 ^‡^ (17.4)
**Odds ratio (95% CI)**	NP		2.23 (0.83–6.06)		0.52 (0.05–3.03)	
*p*-value	NP		0.12		0.68	
**Relative risk reduction,** **% (95% CI)**	62.5 (−32–89)		NP		NP	
*p*-value	0.1944		NP		NP	

* The propensity score analysis set of OS-1 included patients from Denmark and The Netherlands only. ** Data presented for weighted pairs, based on data for 108 women for OS-1 and 42 women for OS-2 (women from the PSAS with hysterectomy prior to matching time were excluded from this analysis). ^†^ Occurrence of invasive procedures after randomization in the RCT, after rFVIIa administration in rFVIIa-exposed patients in the OS, and after time of propensity score-matching in matched control patients from the OS. ^‡^ The number of matched control patients in these groups was not a whole number due to the weighting that was used for the matching process. OS-1, PPH Consortium; OS-2, Bern University Hospital Study. CI, confidence interval; FAS, full analysis set; NP, not pre-specified; PPH, post-partum hemorrhage; PSAS, propensity score-matched analysis set; rFVIIa, recombinant activated factor VII; –, not applicable.

**Table 4:** Thromboembolic events in women with available data from the randomized controlled trial and observational studies.

Study Name	Randomized Controlled Trial (FAS) *		Observational Studies									
			OS-1						OS-2 (FAS)		OS-3 ^‡^ (FAS)	OS-4 (FAS) ^‡‡^
			Denmark (FAS) **		The Netherlands (FAS) ^††^		UK (FAS)					
**Number of women**	rFVIIa N = 51	Ref N = 33	rFVIIa N = 40	No rFVIIa N = 190 ^†^	rFVIIa N = 23	No rFVIIa N = 144	rFVIIa N = 13	No rFVIIa N = 149	rFVIIa N = 52	No rFVIIa N = 113	rFVIIa N = 87	rFVIIa N = 166
**Arterial TEs,** **n (%)**	0	0	0	1 (0.5)	0	1 (0.7)	0	0	0	0	0	1 (0.6) ^§^
**Venous TEs,** **n (%)**	2 (3.9)	0	1 (2.5)	2 (1.1)	1 (4.3)	2 (1.4)	0	4 (2.9)	0	1 (0.9)	0	2 (1.2)
**All TEs,** **n (%)**	2 (3.9)	0	1 (2.5)	3 (1.6)	1 (4.3)	3 (2.1)	0	4 (2.9)	0	1 (0.9)	0	3 (1.8)

rFVIIa-exposed, n = 432; unexposed, n = 629. * Eight women from the reference group were later exposed to rFVIIa (compassionate use) and one received rFVIIa in error, thus the total number of women exposed to rFVIIa was 51. ** For the Danish cohort (OS-1), 1 arterial TE was reported in rFVIIa-exposed women; however, this occurred prior to (15 min) rFVIIa administration and hence was not considered relevant and not included. ^†^ TE data were missing for 9 of the 199 women who were not exposed to rFVIIa in the Danish cohort (OS-1). ^††^ For The Netherlands FAS (OS-1), no data on TEs were available, except for those reported as a complication of embolization procedure, and so data from this cohort were excluded from the meta-analysis. ^‡^ A total of 111 women with PPH were exposed to rFVIIa; however, this included 24 women for whom sPPH was not confirmed (blood loss < 1500 mL or no blood loss information available). ^‡‡^ In this study, two TEs occurred before rFVIIa administration and so were not included in this analysis. ^§^ This arterial TE was an acute myocardial infarction and the woman eventually died secondary to uncontrolled hemorrhages. OS-1, PPH Consortium; OS-2, Bern University Hospital Study; OS-3, UniSeven registry; OS-4, ANZHR; ANZHR, Australian and New Zealand Hemostasis Registry; FAS, full analysis set; PPH, post-partum hemorrhage; rFVIIa, recombinant activated factor VII; Ref, reference group; sPPH, severe post-partum hemorrhage; TE, thromboembolic event.

## Data Availability

Nimes University Hospital is currently implementing a data sharing policy, and any requests for access to data that support the findings of the RCT included in this project will be managed by the Delegation for Clinical Research and Innovation (DRCI). Anonymized patient-level and study-level data from the PPH consortium will be shared upon reasonable request, provided that the requestor has appropriate ethics approval (any requests should be directed to j.g.van_der_bom@lumc.nl in the first instance; data sharing agreements with individual countries may be required for onward data sharing of the consortium data). Study-level data and study protocol underlying the results from Bern University Hospital reported in this article will be shared upon reasonable request, in agreement with the study center’s data sharing policy. Anonymized patient-level, study-level data, and study protocol/case report form underlying the results reported from UniSeven will be shared upon reasonable request. Anonymized patient-level and study-level data from the ANZHR will be shared upon reasonable request, provided that the requestor has appropriate ethics approval. Data will be shared via a Secure eResearch Platform hosted by Monash University (requests should be directed to cameron.wellard@monash.edu).

## Supplementary Materials

The following supporting information can be downloaded at: https://www.mdpi.com/article/10.3390/jcm13092656/s1, Supplementary methods; Supplementary Results; Table S1: Summary of additional study information for the randomized controlled trial and observational studies; Table S2: Patient characteristics in OS-1 and OS-2 (propensity score analysis set); Table S3: Blood transfusions administered before and after rFVIIa administration in the randomized controlled trial and observational studies (full analysis or propensity score analysis sets); Table S4: Sensitivity analysis of primary endpoint (occurrence of invasive procedures) to account for rFVIIa administration after matching time in OS-1 and OS-2 (propensity score analysis set); Table S5: Maternal deaths in women with available data from the randomized controlled trial and observational studies (rFVIIa-exposed, n = 446; unexposed, n = 1717); Figure S1: Definition of matching time for matched patients in relation to sPPH onset (A) and use of matching time in relation to the time window for invasive procedures (panel B); Figure S2: Standardized bias plot for propensity score-matching in (A) OS-1 and (B) OS-2; Figure S3: Proportion of women with any invasive procedure after randomization in the randomized controlled trial by fibrinogen plasma level at baseline (full analysis set); Figure S4: Number of doses of rFVIIa received by women with sPPH in the randomized controlled trial and observational studies (full analysis set); Figure S5: Median dosage of rFVIIa administered in the randomized controlled trial (OS-2, OS-3, and OS-4 (full analysis set)) [17,18,21,22,23,31,32,33,34,35].
