# Supplementary material for: Efficacy and Safety Analyses of Recombinant Factor VIIa in Severe Post-Partum Hemorrhage
Source: J Clin Med. 2024 May 1;13(9):2656. doi: 10.3390/jcm13092656 (PMC11084845; doi:10.3390/jcm13092656)
Supplement: Supplementary file 1 [file jcm-13-02656-s001.zip › jcm-2916067-supplementary.pdf]

Supplementary Material

Contents

Methods 3

S1.1 Study designs 3

S1.1.1 Randomized controlled trial 3
S1.1.2 Observational studies 3
OS-1 3
OS-1 Danish cohort 3
OS-1 Dutch cohort 3
OS-1 UK cohort 3
OS-2 3
OS-3 4
OS-4 4

S1.2 Timescale for primary endpoint analysis 4

S1.3 Safety endpoints 4

S1.4 Statistical analysis 4

S1.4.1 Propensity score matching (OS-1 and OS-2) 4
S1.4.2 Primary endpoint sensitivity analyses (OS-1 and OS-2) 5

Results 5

S2.1 Propensity score matching (OS-1 and OS-2) 5

S2.2 Efficacy of rFVIIa for the treatment of sPPH 6

S2.2.1 Primary endpoint subgroup analysis (RCT) 6
S2.2.2 Primary endpoint sensitivity analysis (OS-1 and OS-2) 6

S2.3 Safety of rFVIIa for the management of sPPH 6

S2.3.1 Thromboembolic events 6
S2.3.2 Deaths 6

S2.4 rFVIIa dosing across all studies 6

References 8

Supplementary Tables and Figures 9

Table S1. Summary of additional study information for the randomized controlled trial and observational studies 9

Table S2. Patient characteristics in OS-1 and OS-2 (propensity score analysis set) 12

Table S3. Blood transfusions administered before and after rFVIIa administration in the randomized controlled trial and observational studies (full analysis or propensity score analysis sets) 14

Table S4. Sensitivity analysis of primary endpoint (occurrence of invasive procedures) to account for rFVIIa administration after matching time in OS-1 and OS-2 (propensity score analysis set) 15

Table S5. Maternal deaths in women with available data from the randomized controlled trial and observational studies (rFVIIa exposed, n=446; unexposed, n=1,717) 16

Figure S1. Definition of matching time for matched patients in relation to sPPH onset (A) and use of matching time in relation to the time window for invasive procedures (B) 17

Figure S2. Standardized bias plot for propensity score matching in (A) OS-1 and (B) OS-2* 18

Figure S3. Proportion of women with any invasive procedure after randomization in the randomized controlled trial by fibrinogen plasma level at baseline (full analysis set) 19

Figure S4. Number of doses of rFVIIa received by women with sPPH in the randomized controlled trial and observational studies (full analysis set) 20

Figure S5. Median dosage of rFVIIa administered in the randomized controlled trial, OS-2, OS-3 and OS-4 (full analysis set) 21
Collaborators 22

Methods

S1.1 Study designs

S1.1.1 Randomized controlled trial

The randomized controlled trial (RCT) was an open-label, parallel-group trial conducted at seven sites in France and one site in Switzerland that originally assessed reduction in the requirement for invasive second-line therapies in severe post-partum hemorrhage (sPPH).[1] Women were randomized 1:1 to receive treatment with either recombinant activated factor VII (rFVIIa) in addition to standard of care or to no concurrent treatment (standard of care alone). Women aged ≥18 years delivering at >27 weeks gestational age were included if they experienced severe hemorrhage at delivery (irrespective of mode of delivery and defined as either blood loss >1,500 mL measured in the graduated bag and/or hemodynamically unstable and/or need for packed cells transfusion) and if sulprostone failed to control bleeding. Additional inclusion criteria have been described previously,[1] and exclusion criteria included history of venous or arterial thrombosis that could contraindicate treatment with rFVIIa. Additional information can be found in **Table S1**.

S1.1.2 Observational studies

The four observational studies are detailed below and additional information can be found in **Table S1**.

OS-1

The PPH consortium (observational study 1 [OS-1]) was a multi-center, retrospective cohort study that included data from the medical records of women with an event of sPPH or persistent PPH (Dutch cohort) from Denmark (2001–2009), The Netherlands (2011–2013) and the UK (2012–2013).

OS-1 Danish cohort

The Danish cohort included women with a birth recorded in the Danish Medical Birth Registry and the Danish Transfusion Database who received ≥10 units of red blood cells (RBC) within 24 h, with additional data extracted from patient chart records as neccessary.[2]

OS-1 Dutch cohort

Women were eligible for inclusion in the Dutch cohort if they had experienced sPPH and had received at least one of the following: ≥4 units RBCs, multicomponent blood transfusion (RBC and fresh frozen plasma [FFP] and or/platelet concentrates), or plasma in addition to RBCs. The Dutch cohort of women with sPPH comprised patients with at least 1,000 mL blood loss refractory to first-line interventions to control bleeding. In this cohort, first-line interventions depended on the cause of bleeding, as previously described.[3] Time of initiation of the first-line intervention to stop PPH was regarded as the moment of diagnosis of persistent PPH, under the assumption that refractoriness to first-line treatment would become evident shortly after initiation of this therapy. Women were followed up from onset until cessation of PPH.

Data for the Dutch cohort were collected through TeMpOH-1, a national retrospective cohort study that included 75% of all hospitals in The Netherlands.[4] Women were identified using records from blood transfusion services and birth registries of participating hospitals, and data were collected from medical records, including information regarding medical history, current pregnancy, causes of hemorrhage, hematological parameters, blood components, fluid resuscitation, and surgical and medical management.[3]

OS-1 UK cohort

Women from the UK were included if they had received ≥8 units RBCs transfused within 24 h and were of ≥20 weeks of gestation. UK data were derived from a national surveillance system (The UK Obstetric Surveillance System) used to identify cases of massive transfusion from obstetric units, with reporters collecting data anonymously from medical records using a data collection form.[5]

OS-2

A single-center, retrospective, observational study was conducted at Bern University Hospital to assess the use of rFVIIa in women with sPPH between 2006–2016 (OS-2).[6] Data were obtained through review of patient chart and electronic medical records, and women with continuous bleeding of ≥1,500 mL within 24 h of delivery were included. Due to some of the recorded data points only having partial timing details available, general rules were

defined to specify the order and timing of events that occurred within the same operation in both rFVIIa-exposed and non-exposed women. Although there is a possibility that the imputations derived from applying these rules may not reflect the actual sequence of events for all cases, these were applied in order to utilize the available data in the most efficient way possible, by defining the most likely sequence of events.

OS-3

UniSeven was a retrospective, multinational, observational data registry that recorded data on the off-label use of rFVIIa between 2003–2014 in the Czech Republic, Slovakia, Slovenia and Hungary (OS-3).[7] Anonymized patient data were collected via a web-based data capture system with secured access for women from the Czech Republic listed with a documented blood loss of ≥1,500 mL within 24 h due to sPPH prior to first dose of rFVIIa.

OS-4

The Australian and New Zealand Haemostasias Registry (ANZHR) documented off-label use of rFVIIa in women that did not have congenital hemophilia across 96 participating hospitals in Australia and New Zealand from 2000–2009 (OS-4).[8,9] Patients were identified by local investigators using blood bank and/or pharmacy records and data were collected using a web-based system with restricted access. Data were included in the updated analysis presented here for women exposed to rFVIIa due to sPPH defined as an obstetric case of hemorrhage with a registered delivery, and women were excluded if delivery took place at <24 weeks of gestational age.

S1.2 Timescale for primary endpoint analysis

For the RCT, the primary endpoint was evaluated at any time point after randomization until end of PPH. For OS-1 and OS-2, this was evaluated within 20 min–24 h following matching time. The 20-minute lag time was implemented to ensure that any invasive procedures included in the analysis resulted from treatment decisions taken after awaiting a possible effect of rFVIIa (based on the typical time [10 min] required to reach peak coagulation effect). Matching time was the timepoint when rFVIIa was administered to exposed women. Controls (not exposed to rFVIIa at the time of matching) were matched to an exposed woman if they had a similar likelihood of receiving rFVIIa (propensity score) at the same time since start of sPPH as the exposed woman. Matching time was then defined in the control to be equivalent to their exposed counterpart (**Figure S1**).

S1.3 Safety endpoints

For the RCT, occurrence of TEs and maternal deaths were recorded from time of inclusion in the protocol up to 5 days postpartum. In OS-1 (UK cohort) and OS-2, these were recorded up until end of hospitalization or death. In the Danish cohort of OS-1, TEs and maternal deaths were recorded up to 6 months after delivery. In the Dutch cohort of OS-1, the timeframe for recording maternal deaths was until end of bleeding, and TEs were only recorded if they occurred as a complication of embolization. OS-3 captured information on TEs and maternal deaths from first rFVIIa administration until end of hospitalization, and for OS-4, data were collected up until 28 days from first rFVIIa administration.

S1.4 Statistical analysis

A post-hoc subgroup analysis was performed for the primary endpoint in the RCT to compare women with baseline fibrinogen plasma levels <2 g/L and ≥2 g/L within both treatment groups.

Analyses comparing bleeding rates (speed of bleeding) before and after administration of rFVIIa were not performed, as the number of patients with more than two estimates of volume of blood loss both before and after administration of rFVIIa was too low (more than two consecutive estimates of volume of blood loss would be required to reliably estimate the bleeding rate).

S1.4.1 Propensity score matching (OS-1 and OS-2)

The purpose of the propensity score matching algorithm was to counteract the confounding by indication present between women who were exposed or not exposed to rFVIIa in OS-1 and OS-2. This process selected two subgroups of women with a comparable course of PPH up to the exposure/matching time, obtained by a separate model of the likelihood of receiving rFVIIa as a function of PPH-related events over time (Cox proportional hazard model with time dependent covariates).[10] Women were ineligible for propensity score matching if they

had pre-planned hysterectomy, hysterectomy before sPPH onset, rFVIIa before sPPH onset or rFVIIa after end of bleeding. For OS-1, data from the UK cohort were analyzed separately, and were not included in the propensity score matching process as this cohort did not have information on time-dependent variables. Detailed information on clinical variables that changed during the course of PPH was available for the Dutch and Danish cohorts.

In both OS-1 and OS-2, the propensity score matching process was performed without knowledge of the primary endpoint, and was stratified for mode of delivery. Pre-specified desired boundaries for standardized bias of −20 to +20 were assigned to account for the expected differences between the two groups. Characteristics considered to be potential variables for the association between use of rFVIIa and treatment outcome, or characteristics considered to be risk factors for the occurrence of the primary outcome measure were selected based on clinical reasoning and prior knowledge with the help of experts within the field. For OS-1, one common model was developed, but patients were matched within each country. The variables included in the propensity score model for OS-1 were cumulative blood loss, cumulative number of invasive procedures (and tamponade balloon), cumulative FFP administration, cumulative crystalloid/colloid administration, first use of platelet concentrate, first use of fibrinogen and primary cause of bleeding. In OS-2, cumulative RBC, cumulative blood loss, cumulative number of previous invasive procedures (invasive procedures and balloon), cumulative FFP administration, and cumulative crystalloids and colloids administration were included as variables.

S1.4.2 Primary endpoint sensitivity analyses (OS-1 and OS-2)

In the propensity score analysis for the primary endpoint in both OS-1 and OS-2, some of the selected matched controls had later exposure to rFVIIa. Administration of rFVIIa after matching time in matched controls could potentially decrease the number of invasive procedures for these patients (if rFVIIa showed a positive treatment effect) and thereby introduce bias (less or no efficacy of rFVIIa). The sensitivity analysis for OS-1 (Dutch cohort only due to availability of data) and OS-2 counted rFVIIa administration following matching time as if an invasive procedure was conducted, in order to see what could have happened if the site did not have access to rFVIIa for this patient (with the assumption that the patient would have an invasive procedure). The endpoint was defined as either an invasive procedure within 20 min–24h of rFVIIa administration, or treatment with rFVIIa after matching time in both the matched controls and in the rFVIIa-exposed group (for any subsequent doses). The results of the sensitivity analyses must be interpreted only in the context of the primary propensity score analysis. Presuming that the matched control patients with rFVIIa administration after matching time would have undergone an invasive procedure with a high probability (in the hypothetical case described above), the treatment effect would be closer to the sensitivity analysis results, but if the patients were less likely to have an invasive procedure then the treatment effect would be closer to the primary analysis result.

Results

S2.1 Propensity score matching (OS-1 and OS-2)

A total of 59 out of 115 rFVIIa-exposed women across OS-1 and OS-2 could not be matched to any of the non-exposed women. In a few cases, women with time-varying data that were so limited that reliable imputations were not feasible were also excluded from matching (none in OS-1 and nine women in OS-2). For the Danish and Dutch cohorts of OS-1, 75.5% (40/53) of women exposed to rFVIIa and eligible for the matching process were matched to 115 controls. There was a good balance between rFVIIa-exposed women and matched controls, with standardized bias within ±20% for all parameters except for primary cause of bleeding (**Figure S2A**), and the two groups were generally similar in terms of baseline characteristics (**Table S2**).

In OS-2, 18 (45%) of the 40 women who were exposed to rFVIIa and eligible for matching were matched to 43 controls using the propensity score model. Although this proportion was less than planned in the study protocol (≥50%), propensity score matching resulted in an acceptable balance between groups, with the standardized bias for most variables within ±20 (**Figure S2B**); however, the results should be interpreted with caution. In the propensity score analysis set, rFVIIa-exposed women and matched controls had generally similar characteristics at baseline (**Table S2**), with some notable differences in the proportion of women with singleton births (72.2%

and 91.2% in matched rFVIIa-exposed women and matched controls, respectively) and those with uterine atony as the primary cause of PPH (72.2% of matched rFVIIa-treated women versus 46.8% of matched controls).

S2.2 Efficacy of rFVIIa for the treatment of sPPH

S2.2.1 Primary endpoint subgroup analysis (RCT)

A post-hoc subgroup analysis of the primary endpoint showed that amongst women with a baseline fibrinogen plasma level ≥2 g/L, invasive procedures occurred in 33% (9/27) of rFVIIa-exposed women versus 94% (31/33) of those in the reference group. For those with a baseline fibrinogen plasma level <2 g/L, invasive procedures occurred in 88% (7/8) of rFVIIa-exposed women versus 100% (5/5) in the reference group (**Figure S3**).

S2.2.2 Primary endpoint sensitivity analysis (OS-1 and OS-2)

In this analysis, the endpoint was defined as either an invasive procedure within 20 min–24h of rFVIIa administration, or treatment with rFVIIa after matching time, in order to account for later exposure to rFVIIa in some of the matched controls (nine women in OS-1 and 21 women in OS-2). The adjusted OR between rFVIIa-exposed and weighted matched controls was 3.17 (95% CI:1.08–10.60; p=0.03) for OS-1 (Dutch cohort) and 0.07 (95% CI: 0.00–0.49; p=0.002) for OS-2 (**Table S4**).

S2.3 Safety of rFVIIa for the management of sPPH

S2.3.1 Thromboembolic events

Of the seven TEs reported in women exposed to rFVIIa, one was an arterial TE (ATE) of acute myocardial infarction in OS-4; this woman died of uncontrolled bleeding. The other six TEs were non-fatal venous TEs (VTE), including one event of short segmental thrombosis of ovarian vein in one woman; deep vein thrombosis in lower limb and pulmonary embolism in lower lobe of right lung in one woman, reported by the investigator as one event; one event of pulmonary embolism, location not reported; one event of deep vein thrombosis, location not reported; one event of pulmonary embolism, location not reported; and one event where full details were not reported. A total of 11 TEs occurred in the group of women who were not exposed to rFVIIa, including one VTE in OS-2, and eight VTEs and two ATEs in OS-1.

S2.3.2 Deaths

Maternal deaths in women with available data from the randomized controlled trial and observational studies are summarized in **Table S5**. One death was recorded in the exposed group of OS-3; however, sPPH was unconfirmed for this woman as her blood loss before rFVIIa administration was unreported, hence she was not included in the full analysis set. One of the 15 deaths reported in rFVIIa-exposed women across all studies was due to complications from thrombolysis of a massive pulmonary embolism (with the massive embolism having occurred before rFVIIa administration), and one rFVIIa-exposed woman had an acute myocardial infarction and died due to causes secondary to uncontrolled hemorrhage. In both of these cases, the death was assessed as unlikely to be related to rFVIIa by a study clinician. The cause of death for 9 of the exposed women was not related to a TE (causes included shock, sepsis, post-operative complications, secondary to uncontrolled hemorrhage, cerebral ischemia, cardiac arrest, multiple organ failure, and uncontrolled blood loss) and unknown or not available for 4 women. Of the 9 deaths that were reported among the 1,717 women in the reference groups across all studies, 4 of the women did not experience a TE (cause of death was amniotic fluid embolism for 1 woman and unknown for 3 women) and data were unavailable for the 5 deaths that occurred in non-exposed women from The Netherlands (OS-1).

S2.4 rFVIIa dosing across all studies

All rFVIIa-exposed women in the RCT received one dose (median=58 µg/kg) in a single slow intravenous injection (2 mL/min; **Figure S4** and **Figure S5**). The majority of women in the observational studies (264/382) received one dose (across all studies except UK patients from OS-1, for whom data were unavailable. Data from the Danish cohort of OS-1 were listed as missing.). The proportion of women who received ≥2 doses ranged from 12%–28% (**Figure S4**).

Median first dose of rFVIIa in the full analysis set ranged from 63 µg/kg in OS-2 to 105 µg/kg in OS-4 (**Figure S5**). Median second and third doses (for those who received ≥1 dose) ranged from 73–100 µg/kg across studies. In OS-1 only the total rFVIIa dose in mg was recorded, with median total dose ranging from 5–6 mg amongst the cohorts.

References

1. Lavigne-Lissalde, G.; Aya, A.G.; Mercier, F.J.; Roger-Christoph, S.; Chauleur, C.; Morau, E.; Ducloy-Bouthors, A.S.; Mignon, A.; Raucoules, M.; Bongain, A.; et al. Recombinant human FVIIa for reducing the need for invasive second-line therapies in severe refractory postpartum hemorrhage: A multicenter, randomized, open controlled trial. J. Thromb. Haemost. 2015, 13, 520–529. https://doi.org/10.1111/jth.12844.
2. McKinnon Edwards H. Aetiology and treatment of severe postpartum hemorrhage. *Dan Med J.* **2018,** *65* (3), B5444.
3. Henriquez DDCA, Gillissen A, Smith SM, et al. Clinical characteristics of women captured by extending the definition of severe postpartum hemorrhage with ‘refractoriness to treatment’: a cohort study. *BMC Pregnancy and Childbirth*. **2019**, *19,* 361.
4. Henriquez, D.; Caram-Deelder, C.; le Cessie, S.; Zwart, J.J.; van Roosmalen, J.J.M.; Eikenboom, J.C.J.; So-Osman, C.; van de Watering, L.M.G.; Zwaginga, J.J.; Koopman-van Gemert, A.; et al. Association of timing of plasma transfusion with adverse maternal outcomes in women with persistent postpartum hemorrhage. JAMA Netw. Open 2019, 2, e1915628. https://doi.org/10.1001/jamanetworkopen.2019.15628.
5. UKOSS. Massive transfusion in major obstetric hemorrhage. Available online: https://www.npeu.ox.ac.uk/ukoss/completed-surveillance/mtmoh (accessed on 12 Feb 2024).
6. Colucci, G.; Helsing, K.; Biasiutti, F.D.; Raio, L.; Schmid, P.; Tsakiris, D.A.; Eberle, B.; Surbek, D.; Lämmle, B.; Alberio, L. Standardized management protocol in severe postpartum hemorrhage: A single-center study. Clin. Appl. Thromb./Hemost. 2018, 24, 884–893. https://doi.org/10.1177/1076029618758956.
7. Blatny, J.; Seidlova, D.; Penka, M.; Ovesna, P.; Brabec, P.; Sevcik, P.; Ventruba, P.; Cerny, V. Severe postpartum haemorrhage treated with recombinant activated factor VII in 80 Czech patients: Analysis of the UniSeven registry. Int. J. Obstet. Anesth. 2011, 20, 367–368. https://doi.org/10.1016/j.ijoa.2011.07.008.
8. Phillips, L.E.; McLintock, C.; Pollock, W.; Gatt, S.; Popham, P.; Jankelowitz, G.; Ogle, R.; Cameron, P.A.; Australian; New Zealand Haemostasis, R. Recombinant activated factor VII in obstetric hemorrhage: Experiences from the Australian and New Zealand Haemostasis Registry. Anesth. Analg. 2009, 109, 1908–1915. https://doi.org/10.1213/ANE.0b013e3181c039e6.
9. Zatta A, McQuilten Z, Kandane-Rathnayake R, et al. The Australian and New Zealand Haemostasis Registry: ten years of data on off-licence use of recombinant activated factor VII. *Blood Transfus*. **2015**, *13* (1), 86–99.
10. Lu B. Propensity score matching with time-dependent covariates. Biometrics. 2005, 61 (3), 721–728.

Supplementary Tables and Figures

Table S1. Summary of additional study information for the randomized controlled trial and observational studies

	Randomized controlled trial	Observational studies					
		OS-1			OS-2	OS-3	OS-4
		Denmark	Netherlands	UK			
**Aim**	To evaluate the interest in using rFVIIa in the treatment of severe PPH unresponsive to conventional treatment and to define, if applicable, the number of interventional embolization procedures, vascular ligations, hysterectomies and transfusions prevented due to the early use of rFVIIa.	To compare the occurrence of any invasive procedure after first treatment with rFVIIa with the occurrence of any invasive procedure without treatment with rFVIIa in a propensity score-matched population of women with severe PPH.				To describe the proportion of women with an event of severe PPH that avoided invasive procedures following treatment with rFVIIa.	
**Study population**	Women with severe PPH could be included in the trial if sulprostone had failed to control their bleeding.	Data were identified and collected using the Danish Medical Birth Registry (DMBR) and the Danish Transfusion Database (DTD). The DMBR collects information on all births in Denmark and this includes clinical and demographic data from each birth. The DTD is a national database and	TeMpOH-1 was a national retrospective cohort study in the Netherlands, which included 75% of all hospitals. Data were collected from medical records and included information about previous medical history, current pregnancy, causes of hemorrhage, hematological parameters, blood components, fluid resuscitation and	Data from UK comes from a national surveillance system (The United Kingdom Obstetric Surveillance System UKOSS) used to identify cases of massive transfusion from consultant-led obstetric units nationally in the UK. Reporters were asked to complete a data collection form using the medical records of the women.	Women admitted to Bern University Hospital, Bern, Switzerland. during the study period for each cohort, either as the primary hospital or secondary hospital (transfer patients).	UniSeven was a retrospective observational data registry to collect data on various off-label use of rFVIIa from people from the Czech Republic, Slovakia, Slovenia and Hungary. The current study included data only on women with event of severe PPH from the Czech Republic as this subset of data had undergone data quality control.	Women from Australia and New Zealand with an event of sPPH, treated with rFVIIa due to a critical bleed and were registered in the Australian and New Zealand Haemostasias Registry (ANZHR) documenting off-label rFVIIa use.

		contains information of the quantity and type of blood products transfused and the time of each transfusion.	surgical and medical management.				
**Excluded**	No exclusions; 84 women were included and all were considered to have completed the trial.	40 exposed women were included in the FAS, 3 were excluded with a hysterectomy before severe PPH and 14 with a hysterectomy before rFVIIa. Of the 23 exposed patients, 16 were matched to controls.	37 exposed women were included in the FAS, of these, 7 were excluded due to hysterectomy before rFVIIa. Of the 30 exposed patients, 24 were matched to controls.	13 women received rFVIIa; 1 woman had a hysterectomy before rFVIIa, 2 had unknown timing of hysterectomy, 5 had any invasive procedures after rFVIIa.	52 exposed women were included in the FAS; of these, 12 were not eligible for propensity score matching. Of the 40 eligible, 22 were not matched. A final of 18 exposed patients were matched for primary analysis.	44/87 women included in the FAS were excluded from the primary endpoint analysis due to hysterectomy before or conjoint with rFVIIa administration. The population at risk of further invasive procedures included 43 women.	11/177 women treated with rFVIIa were excluded because gestational age was <24 weeks. 92/166 women included in the FAS were excluded from primary analysis due to hysterectomy before/concurrent with rFVIIa administration (54), or missing timing of invasive procedure (38).
**rFVIIa not as last resort**	In the exposed arm, women received rFVIIa at time of randomization (after failure of sulprostone).	Not specified.			Not specified.	Not specified.	Not specified.
**Indication for rFVIIa**	Failure of sulprostone to control bleeding.	Not specified, however, it was generally late in the course of PPH (mean ~6 h after severe PPH onset for the FAS). This suggests that rFVIIa was used in the most severe clinical situations.			Cohort 1: Persistent massive bleeding after 8 U RBCs and 4 U FFP; cohort 2: at discretion of treating team; cohort 3/4: persistent bleeding after mechanical, physiological and pharmacological measures (uterotonics, fluids, TXA).	Not specified, however, the mean time from start of bleeding to first rFVIIa dose was 409.9 minutes (~6.8 h, range: 30 to 3600 minutes).	Not specified, but off-label use is often as last resort.

**Lab tests performed at baseline**	Fibrinogen plasma level ≥2 g/L: 27/42 (64.3%) rFVIIa-exposed women (data missing for 7/42) and 33/42 (78.6%) non-exposed women (data missing for 4/42).<br><br>Platelets ≥50x10^{9}/L: 38/42 (90.5%) rFVIIa-exposed women (data missing for 3/42) and 41/42 (97.6%) non-exposed women (data missing for 1/42).<br><br>pH not reported.	Not reported.	Fibrinogen plasma level ≥2 g/L: 10/22 (45.5%) for the rFVIIa group overall.<br><br>Platelets ≥50x10^{9}/L: 19/22 (76.0%) for the rFVIIa group overall.<br><br>pH ≥7.2: 17/22 (94.4%).	Fibrinogen plasma level ≥2 g/: 27/87 (31.0%) women (data missing for 3/87).<br><br>Platelets ≥50x10^{9}/L: 73/87 (83.9%) women.<br><br>pH ≥7.2: 74/87 (85.1%) women (data missing for 9/87).	Fibrinogen plasma level ≥2 g/L: 42/166 (25.3%) women (data missing for 33/166).<br><br>Platelets ≥50x10^{9}/L: 131/166 (78.9%) women (data missing for 14/166).<br><br>pH ≥7.2: 84/166 (50.6%) women (data missing for 52/166).

OS-1, PPH Consortium; OS-2, Bern University Hospital Study; OS-3, UniSeven registry; OS-4, ANZHR.

ANZHR, Australian and New Zealand Hemostasis Registry; FAS, full analysis set; FFP, fresh frozen plasma; h, hours; PPH, post-partum hemorrhage; RBC, red blood cells; rFVIIa, recombinant activated factor VII; U, units.

Table S2. Patient characteristics in OS-1 and OS-2 (propensity score analysis set)

	Observational studies			
	OS-1*		OS-2	
**Baseline characteristics**	**rFVIIa N=40**	**Matched controls** N=40**	**rFVIIa N=18**	**Matched controls** N=18**
**Age at delivery, years**				
Median (IQR)	31.5 (27–35.5)	32 (29–36)	–	–
**Maternal body weight, kg*****				
Median (IQR)	–	–	70.0 (63.0 - 89.5)	70.0 (63.1 - 80.0)
**Cause of PPH, n (%)**†				
AIP	6 (15.0)	3.50 (8.8)	4 (22.2)	5.0 (27.8)
Placental abruption	2 (5.0)	2.67 (6.7)	1 (5.6)	2.3 (12.5)
Placental retention	3 (7.5)	4.42 (11.0)	0	0
Trauma‡	3 (7.5)	4.58 (11.5)	0	1.3 (7.4)
Uterine atony	23 (57.5)	24.08 (60.2)	13 (72.2)	8.4 (46.8)
Other	3 (7.5)	0.75 (1.9)	0	1.0 (5.6)
**Delivery type, n (%)**				
Caesarean section	16 (40.0)	16 (40.0)	16 (88.9)	16 (88.9)
**Multiple birth (≥2), n (%)**				
Yes	2 (5.0)	1.83 (4.6)	5 (27.8)	1.6 (8.8)
**Invasive procedure(s) prior to matching time, n (%)**††				
Any	9.1 (22.71)	7 (17.5)	11 (61.1)	7.0 (38.9)
Hysterectomy	0	0	0	0
**Blood loss**§				
Blood loss (L), mean (SD)	5.53 (1.39)	5.64 (1.46)	3.02 (1.15)	2.84 (0.75)
Blood loss (log scale), mean (SD)	1.71 (0.33)	1.73 (0.38)	–	–
**Preeclampsia/eclampsia/HELLP, n (%)**				
Yes	6 (15.00)	6.42 (16.04)	2 (11.1)	0.8 (4.2)
No	34 (85.00)	33.58 (83.96)	16 (88.9)	17.3 (95.8)
**Platelets prior to matching time, units**				
n	33.0	84.0	4	3.5
Mean (SD)	1.39 (2.29)	1.14 (1.34)	1.0 (0.0)	1.0 (0.0)
**Total colloids, mL**				
n	38.0	110.0	15	16.8

Mean (SD)	2000.0 (1071.8)	1677.24 (883.8)	1300.0 (702.0)	1699.0 (661.1)
**Total crystalloids, mL**				
n	39.0	109.0	16	17.4
Mean (SD)	4907.67(3444.9)	5024.3 (3063.6)	2353.1 (1215.1)	3471.8 (1063.0)
**Fibrinogen prior to matching time, n (%)**				
Yes	8 (20.0)	10.4 (26.0)	6 (33.3)	2.6 (14.4)
**Tranexamic acid prior to matching time, n (%)**				
Yes	27 (67.5)	25.0 (62.5)	10 (55.6)	8.0 (44.4)
**Oxytocin prior to matching time, n (%)**				
Yes	32 (80.00)	31.33 (78.33)	16 (88.89)	15.2 (84.44)
**Other uterotonics prior to matching time, n (%)**				
Yes	37 (92.50)	36.17 (90.42)	15 (83.33)	11.9 (66.11)
**Country, n (%)**				
N	40	115	18	43
Denmark	16 (40.0)	44 (38.26)	0	0
The Netherlands	24 (60.0)	71 (61.74)	0	0
Switzerland	0	0	18 (100)	43 (100)

Baseline data for the covariates used for propensity score matching are presented in this table, with the exception of red blood cells and fresh frozen plasma transfusions, which are presented in Table S3.
*Denmark and The Netherlands cohorts only. **In OS-1, 115 patients were matched, and following the weighting process, 40 matched pairs were defined. In OS-2, 43 patients were matched and 18 matched pairs defined. Data are presented here for the weighted groups. ***End of pregnancy weight, adjusted for weight of the baby. †Primary cause of PPH; a woman may have had more than one cause of PPH. ‡Trauma included all cases of trauma, uterine rupture and birth canal injury. ††Data presented for invasive procedures from start of sPPH until 20 minutes before matching time. §Interpolated accumulated blood loss at matching time (numbers are shown as weighted mean and SD to represent the data presented in Figure S5).
OS-1, PPH Consortium; OS-2, Bern University Hospital Study.
AIP, abnormally invasive placenta; HELLP, hemolysis, elevated liver enzymes and low platelets; IQR, interquartile range; OS, observational study; PPH, post-partum hemorrhage; rFVIIa, recombinant activated factor VII; SD, standard deviation.

Table S3. Blood transfusions administered before and after rFVIIa administration in the randomized controlled trial and observational studies (full analysis or propensity score analysis sets)

	Randomized controlled trial (FAS)		Observational studies					
			OS-1* Denmark and the Netherlands (PSAS)		OS-2* (PSAS)		OS-3 (FAS)	OS-4 (FAS)
	rFVIIa N=42	Ref N=42	rFVIIa N=40	Matched controls** N=40	rFVIIa N=18	Matched controls** N=18	rFVIIa N=87	rFVIIa N=166
**Before first rFVIIa dose/randomization/matching time*** **								
**RBC (units), n**	16	11	40	40	16	14.3	87	165
Mean no. of units (SD)	2.7 (1.3)	3.1 (2.3)	6.6 (4.0)	7.5 (4.9)	4.4 (2.9)	4.5 (1.7)	9.1 (6.3)	11.6 (8.4)
**FFP (units), n**	6	6	40	40	14	14.0	87	164
Mean no. of units (SD)	3.3 (2.2)	4.0 (1.1)	4.3 (4.0)	4.2 (3.3)	4.4 (3.0)	4.0 (1.7)	8.8 (6.2)	7.1 (6.1)
**After first rFVIIa dose/randomization/matching time****								
**RBC (units), n**	25	28	40	40	15	16.4	86	159
Mean no. of units (SD)	4.2 (3.0)	4.4 (3.0)	6.9 (5.4)	5.6 (5.5)	7.1 (5.1)	8.1 (3.5)	4.3 (3.3)	5.9 (7.9)
**FFP (units), n**	19	20	40	40	17	14.3	86	156
Mean no. of units (SD)	4.3 (3.5)	4.4 (2.2)	4.1 (3.4)	3.8 (4.9)	5.9 (5.9)	7.2 (3.2)	5.1 (4.4)	4.2 (6.8)

n=no of women receiving a transfusion. *For OS-1 and OS-2, before first rFVIIa dose corresponds to 'before matching time' and after first rFVIIa dose corresponds to 'after matching time', where matching time is defined as time of first administration of rFVIIa. For matched controls, matching time is derived from the matching process and is equal to the period from onset of sPPH to time of first administration of rFVIIa for the patient for which they are a matched control. **In OS-1, 115 patients were matched, and following the weighting process, 40 matched pairs were defined. In OS-2, 43 patients were matched and 18 matched pairs defined. Data are presented here for the weighted groups.
***Randomization refers to the randomized controlled trial only; all other studies from first dose.
OS-1, PPH Consortium; OS-2, Bern University Hospital Study; OS-3, UniSeven registry; OS-4, ANZHR.
ANZHR, Australian and New Zealand Haemostasias Registry; FAS, full analysis set; FFP, fresh frozen plasma; N: Number of (weighted) patients; PSAS, propensity score matched analysis set; RBC, red blood cells; rFVIIa, recombinant activated factor VII; SD, standard deviation.

Table S4. Sensitivity analysis of primary endpoint (occurrence of invasive procedures) to account for rFVIIa administration after matching time in OS-1 and OS-2 (propensity score analysis set)

	Matched controls, n (%)	Matched exposed, n (%)	Number of groups	Odds ratio	95% confidence interval	P value
**OS-1 (The Netherlands)**						
**Any IP or rFVIIa after matching time***	7.3 (31)	15 (63)	24	3.17	1.08–10.60	0.03
**OS-2**						
**Any IP or rFVIIa after matching time***	12.3 (70)	4 (22)	18	0.07	0.00–0.49	0.0019

Matching time was the timepoint at which rFVIIa was administered in exposed women, and for matched controls was equivalent to the same timepoint as the patient with whom they were matched.
*For controls who received rFVIIa after the time of matching and exposed women who received additional doses of rFVIIa, these were imputed as an IP. Nine of 118 in the Dutch cohort of OS-1 and 21 of 42 matched controls in OS-2 received rFVIIa after matching time.
OS-1, PPH Consortium; OS-2, Bern University Hospital Study.
IP, invasive procedure; rFVIIa, recombinant activated factor VII.

Table S5. Maternal deaths in women with available data from the randomized controlled trial and observational studies (rFVIIa exposed, n=446; unexposed, n=1,717)

	Randomized controlled trial (FAS)*		Observational studies										
			OS-1						OS-2 (FAS)		OS-3**		OS-4 (FAS)
			Denmark (FAS)		Netherlands (FAS)		UK (FAS)				All exposed	FAS	
	rFVIIa N=51	Ref N=33	rFVIIa N=40	No rFVIIa N=199	rFVIIa N=37	No rFVIIa N=1223	rFVIIa N=13	No rFVIIa N=149	rFVIIa N=52	No rFVIIa N=113	rFVIIa N=111	rFVIIa N=87	rFVIIa N=166
**Maternal deaths**	0	0	0	2 (1.0)	2 (5.4)	5 (0.4)	0	2 (1.3)	0	0	1 (0.9)	0	13 (7.8)

*8 women from the reference group were later exposed to rFVIIa (compassionate use) and one received rFVIIa in error, thus the total number of women exposed to rFVIIa was 51. **A total of 111 women with PPH were exposed to rFVIIa; however, this included 24 women for whom sPPH was not confirmed (blood loss <1500 mL or no blood loss information available).
OS-1, PPH Consortium; OS-2, Bern University Hospital Study; OS-3, UniSeven registry; OS-4, ANZHR.
ANZHR, Australian and New Zealand Haemostasias Registry; FAS, full analysis set; PPH, post-partum hemorrhage; rFVIIa, recombinant activated factor VII.

Figure S1. Definition of matching time for matched patients in relation to sPPH onset (A) and use of matching time in relation to the time window for invasive procedures (B)

A)

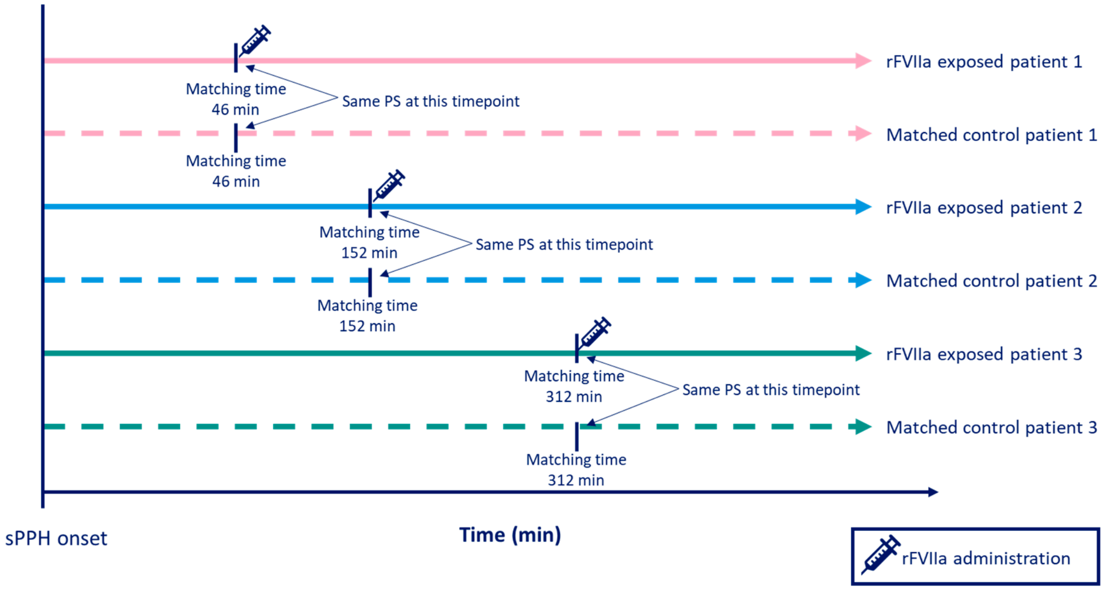

B)

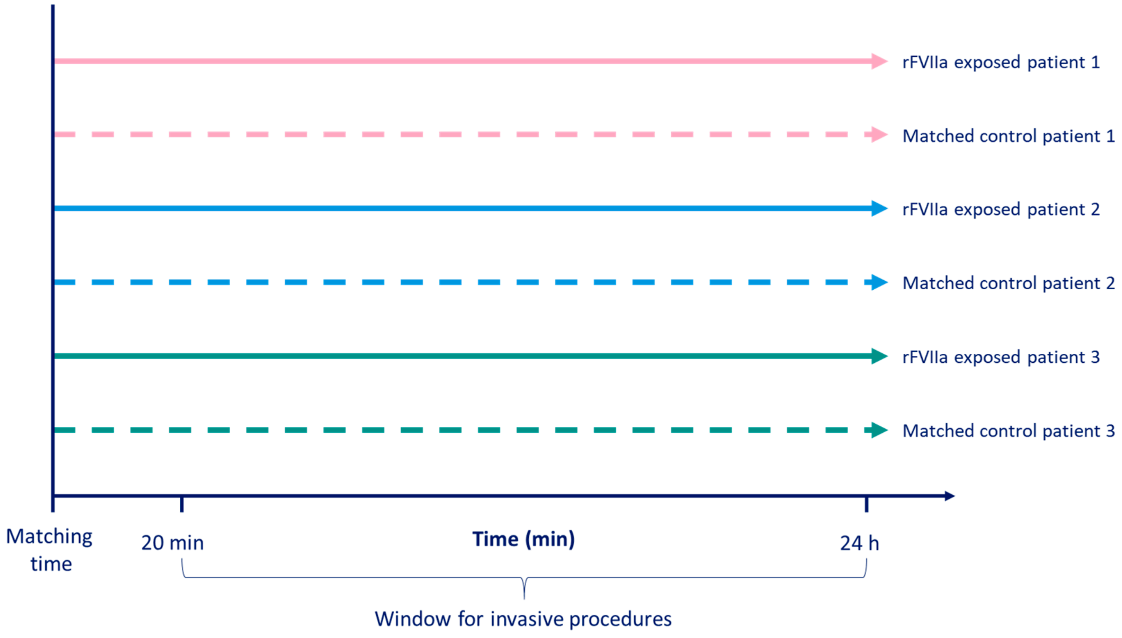

Matching time was the timepoint at which rFVIIa was received in the matched exposed women. Controls (women who were not exposed) were matched to an exposed woman if they had a similar course of PPH and likelihood of receiving rFVIIa (propensity score) at the same timepoint as the exposed woman. Matching time was then defined in the control to be equivalent to their exposed counterpart (A). IPs were evaluated from 20 min–24 h following matching time (B).
IP, invasive procedure; PS, propensity score; rFVIIa, recombinant activated factor VII;
sPPH, severe post-partum hemorrhage.

Figure S2. Standardized bias* plot for propensity score matching in (A) OS-1 and (B) OS-2

A)

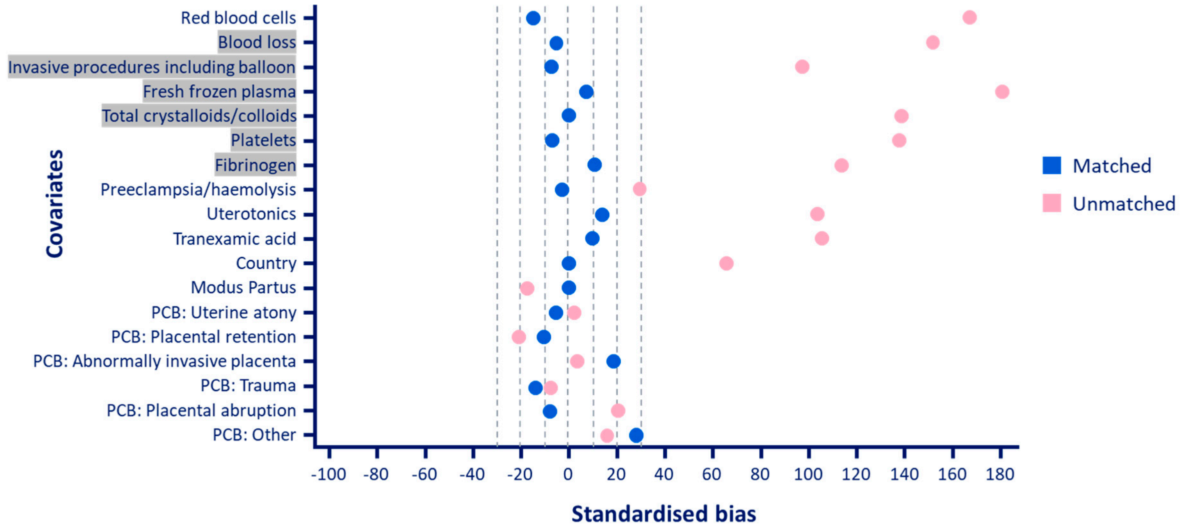

B)

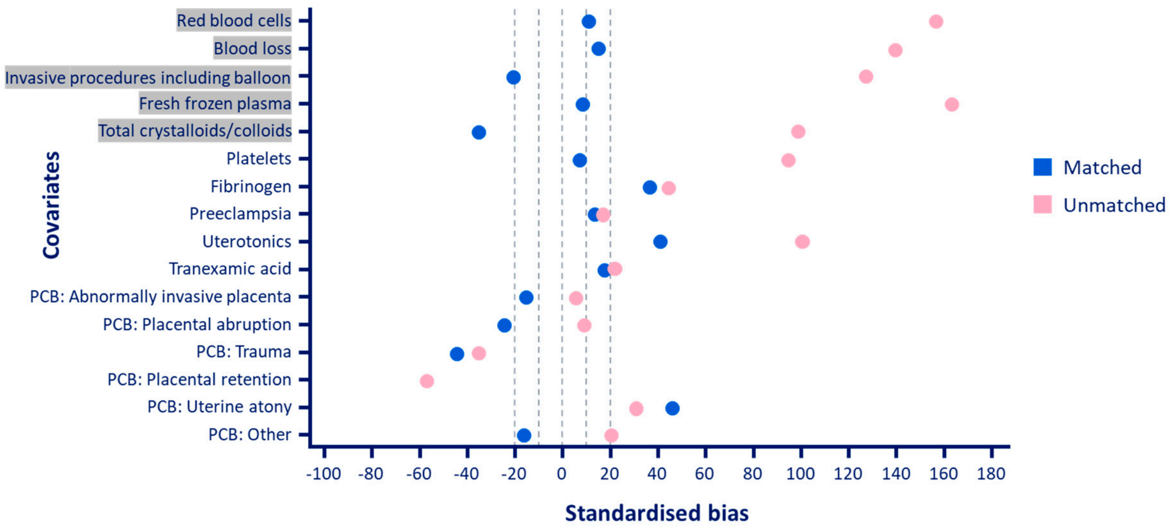

*Standardized bias: Difference in means between groups/within group standard deviation. Covariates that were used for matching are highlighted with grey background. There were some differences in the covariates used between panels A and B due to differences in the study protocols for OS-1 and OS-2. Unmatched data (pink dots) refers to before matching. Matched data (blue dots) lying within the pre-specified bounds for standardized bias of -20 to 20 are considered to be balanced.
OS-1, PPH Consortium; OS-2, Bern University Hospital Study. PCB, primary cause of bleeding.

Figure S3. Proportion of women with any invasive procedure after randomization in the randomized controlled trial by fibrinogen plasma level at baseline (full analysis set)

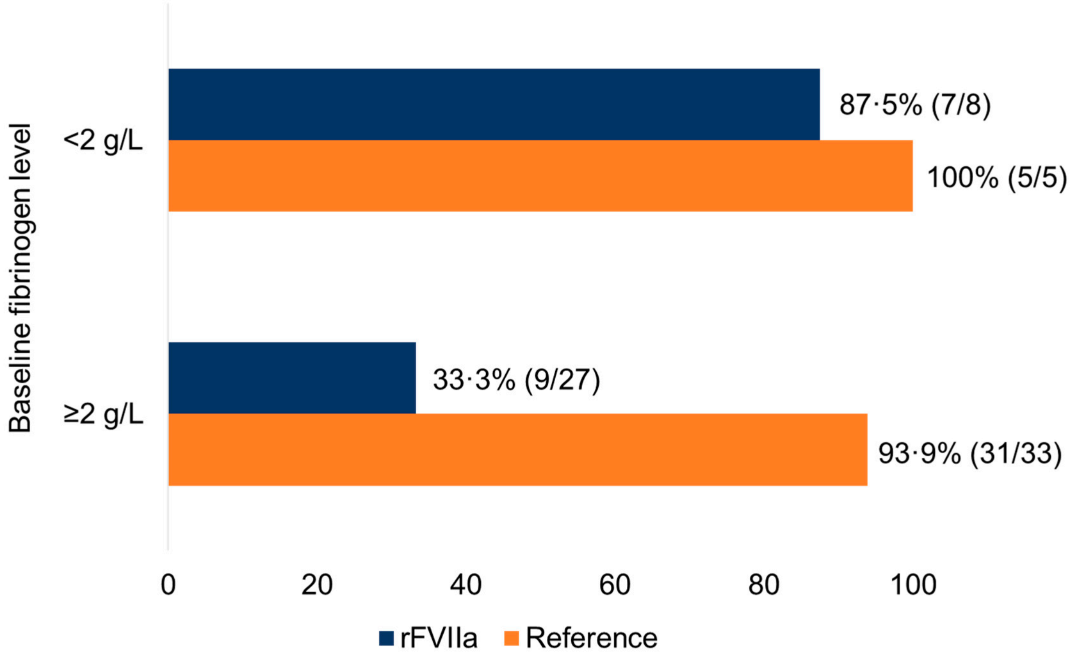

Baseline fibrinogen plasma level data were missing for 7 women in the rFVIIa group and for 4 women in the reference group. rFVIIa, recombinant activated factor VII.

Figure S4. Number of doses of rFVIIa received by women with sPPH in the randomized controlled trial and observational studies (full analysis set)

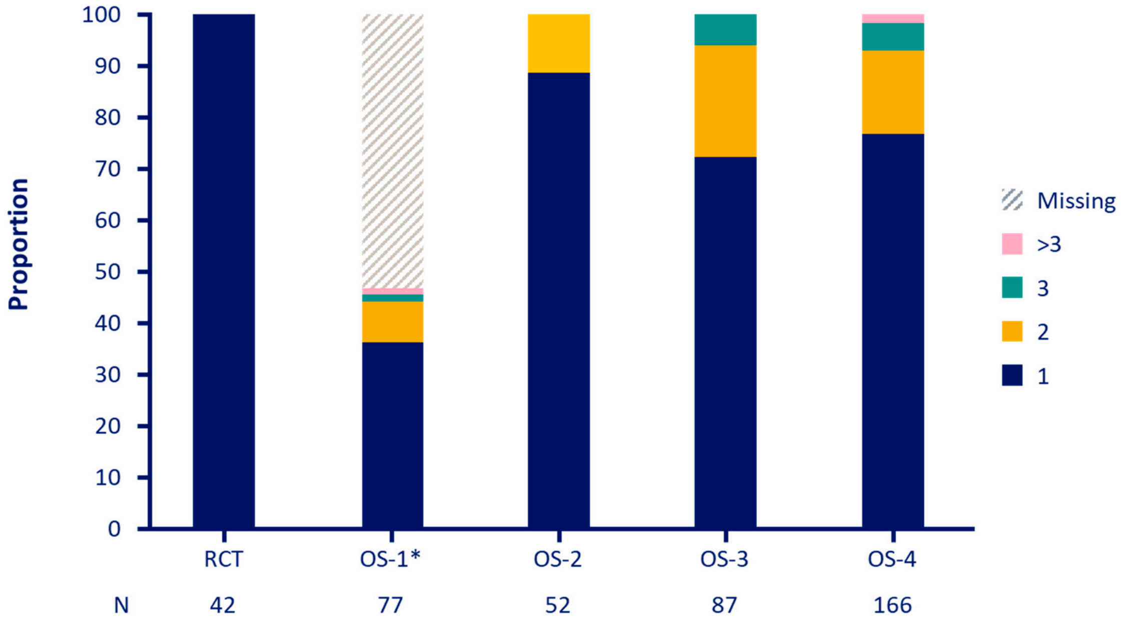

*Danish and Dutch cohorts of OS-1 (data were unavailable for the UK cohort). Data regarding the number of doses of rFVIIa received are shown as missing for the Danish cohort.

OS-1, PPH Consortium; OS-2, Bern University Hospital Study; OS-3, UniSeven registry; OS-4, ANZHR.

ANZHR, Australian and New Zealand Haemostasias Registry; RCT, randomized clinical trial; rFVIIa, recombinant activated factor VII; sPPH, severe post-partum hemorrhage.

Figure S5. Median dosage of rFVIIa administered in the randomized controlled trial, OS-2, OS-3 and OS-4 (full analysis set)

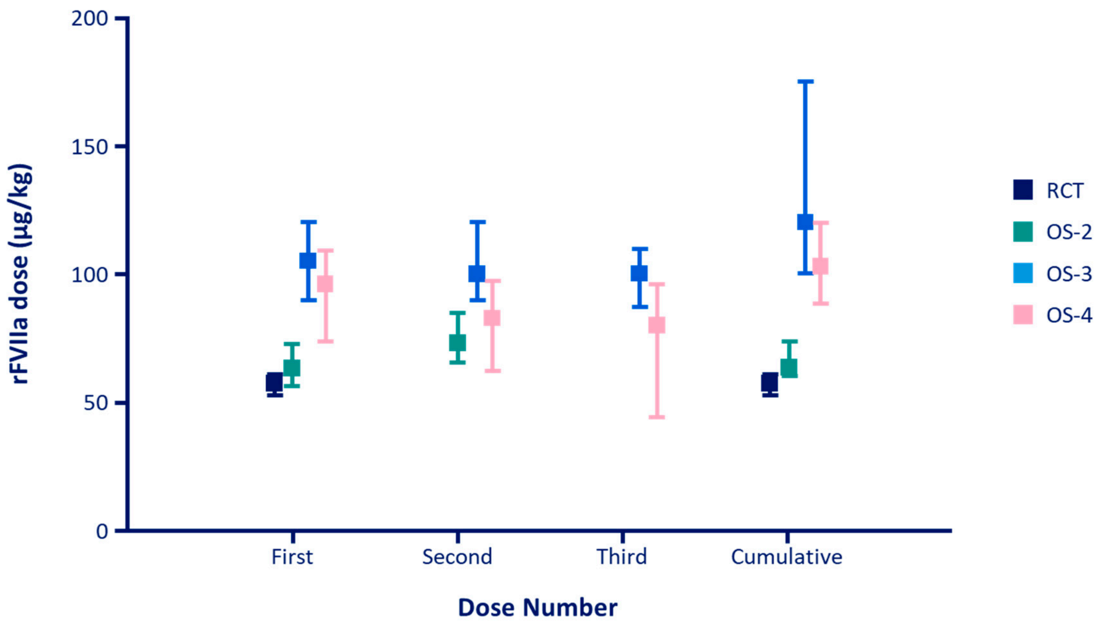

Error bars represent the interquartile range. Data from OS-1 are not presented as information on dose/kg body weight was unavailable.
OS-1, PPH Consortium; OS-2, Bern University Hospital Study; OS-3, UniSeven registry;
OS-4, ANZHR.
ANZHR, Australian and New Zealand Haemostasias Registry; RCT, randomized clinical trial;
rFVIIa, recombinant activated factor VII.

Collaborators

Randomized controlled trial conducted in France and Switzerland (NCT00370877):

Antoine Guy Aya, Françoise Boehlen, André Bongain, Sophie Bouvet, Céline Chauleur, Anne-Sophie Ducloy-Bouthors, Pascale Fabbro-Peray, Jean-Christophe Gris, Alexandre Mignon, Philippe de Moerloose, Estelle Morau, Marc Raucoules, Sandrine Roger-Christoph.

Bern University Hospital:

Annina Etter, Iris Müller.

PPH Consortium:

Catherine Deneux-Tharaux, Anne Wikkelsoe, TeMpOH-1 steering group, TeMpOH-1 research group, additional TeMpOH-1 study contributors, UKOSS Massive transfusion study group.

UniSeven:

Petra Ovesná.

Australian and New Zealand Hemostasis Registry:

Peter Cameron, Scott Dunkley, Andrew Hannaford, Roger Houben, James Isbister, Gary Jankelowitz, John McNeil, Zoe McQuilten, Louise Phillips, Kunnapoj M Pruksawongsin, Hatem Salem, Wendy Thomas, Neil Waters, Cameron Wellard, Erica Wood, Amanda Zatta.

Novo Nordisk:

Ekaterina Gresko, Damaris Bausch-Fluck, Susanna Lövdahl, Jens Magelund Tarp, Alonso Rojas-Rios, Daniel David Rubens.

TeMpOH-1 steering group:

Dacia D. C. A. Henriquez, Saskia le Cessie, Joost J. Zwart, Jos J. M. van Roosmalen, Jeroen C. J. Eikenboom, Cynthia So-Osman, Leo M. G. van de Watering, Jaap Jan Zwaginga, Kitty W. M. Bloemenkamp, Johanna G. van der Bom.

TeMpOH-1 research group:

Thomas van den Akker, Camila Caram-Deelder, Derek van Duuren, Cock M. C. Bank, Yvette S. Snuif–de Lange, Adriaan J. van Gammeren, and Dimitri N. M. Papatsonis, Harriet Klinkspoor, Marjolein Kok, Bauke A. de Boer, Josje Langenveld, Mathie P. G. Leers, Jart H. C. Diris, Rene D. Kok, Paula Engbers, Marjo J. C. P. Hanssen, Wim J. van Wijngaarden, Daniela H. Schippers, Jos W. J. van der Stappen, Tom H. M. Hasaart, Daan H. van de Kerkhof, Jacques B. de Kok, Gijs, A. van Unnik, Wouter Kortlandt, Nico W. E. Schuitemaker, Friso M. C. Delemarre, Hans L. P. van Duijnhoven, Hans J. Duvekot, Sietske Hogenboom, Gunilla Kleiverda, Mylene J. W. van Etten–van Hulst, Karin Pagano Mirani–Oostdijk, Corine van Kampen, Martin J. N. Weinans, Henk J. Adriaanse, Anjoke J. M. Huisjes, Marieke A. M. Frasa, Jeffrey F. W. Keuren, Claudia A. van Meir, Hanneke Feitsma, Francisca Hudig, J. Marko Sikkema, Marleen I. Baas, Michael A. Fouraux, Godfried C. H Metz, Bas W. H. Nij Bijvank, Henk J. M. Rondeel, Jos M. T. Roelofsen, Marjolein Doesburg–van Kleffens, Sander C. de Wit, Hans Versendaal, Floor Weerkamp, Yvonne M. C. Henskens, Liesbeth H. C. J. Scheepers, David P. van der Ham, Jan W. Smit, Fedde van der Graaf, Martina M. Porath, Paulien C. M. van der Salm, Merel van Wijnen, Martina Pontesilli, Frederique M. van Dunné, Gabrielle A. E. Ponjee, Marinka S. Post, Betty S. van der Veen, Josien T. J. Brons, Jennita Slomp, Arjan de Mare, Anja Leyte, Eline S. A. van den Akker, Henri de Wet, Diana M. R. van der Borden, Henk A. Bremer, G. H. Martine Tax, Marjolein J. de Vries, Karin de Boer, Harm de Waard, Ries H. de Keijzer, Jan M. Burggraaff, Jos G. J. Pouwels, Nicolette van Gemund, Lenneke Prinzen, Henriet A. Hendriks, Brenda B. J. Hermsen, Stephan G. A. Koehorst, Tamara E. M. Verhagen, Erik van Beek, Chris M. Hackeng, Peter J. Kabel, Pieternel Steures, Ivo M. A. van Dooren, Etienne C. H. J. Michielse, Helena Chon, Marco Treskes, Harry Visser, Esther Oostenveld, Dick H. M. Peters, Maureen T. M. Franssen, J. Henk Meekers, Mallory D. Woiski, Liesbeth C. M. van Pampus, Martijn A. Oudijk, Karen M.

K. de Vooght, Rolf L. M. Cikot, Leendert J. Mostert, Huib Ceelie, Annemarie M. G. Huijssoon, Christianne J. M. de Groot, Otto Visser, Niels Jonker, Annemarieke Koops, Angelo Hooker, Nada Osmanovic, Herman J. L. M. Ulenkate, Bianca Visschers, Jeffrey F. W. Keuren, Gratia D. M. Martens.

Additional TeMpOH-1 study contributors

Rosanne M. Loeff, Renske J. Van Goeverden, Bram Eijlers, Anne Hillebrand, Saskia E. Spelmink, Tom J. Beunder, Valerie Harskamp, Merlijn Wind, Mark D. Koning, Roos A. Cramer, Artyom Veenstra, Sharissa M. Smith, Elmara E. Ensing, Clara Kolster-Bijdevaate, Marjolein S. Bourgonje-Verhart, Lisanne Bonsen, Cecilia E. Bleeker-Taborh, Ellen Roos-van Milligen, Karin J. van Brussel-de Groot, Ouisam Zouitni.

UKOSS Massive transfusion study group

Laura Green, Marian Knight, Frances Seeney, Cathy Hopkinson, Peter W. Collins, Rachel E Collis, Nigel A.B. Simpson, Andrew Weeks, Simon J Stanworth.
